# Pre-diagnostic beta-blocker use and head- and neck cancer risk: A nationwide population-based case-control study

**DOI:** 10.1097/MD.0000000000016047

**Published:** 2019-06-14

**Authors:** Min-Su Kim, Kyung Do Han, Soon Young Kwon

**Affiliations:** a Department of Otorhinolaryngology-Head and Neck Surgery, CHA Bundang Medical Center, CHA University, Seongnam; b Department of Biostatistics, College of Medicine, The Catholic University of Korea; c Department of Otorhinolaryngology-Head and Neck Surgery, Korea University College of Medicine, Seoul, Republic of Korea.

**Keywords:** adrenergic beta-antagonists, head and neck neoplasm, incidence

## Abstract

β-blockers have been reported to exhibit potential anticancer effects in various cancer studies. However, few clinical studies concerning head and neck cancer have been conducted. We hypothesized that β-blockers could decrease the incidence of head and neck cancer. Therefore, we investigated the association between β-blocker treatment and head and neck cancer incidence.

Between January 2006 and December 2015, we selected 12,127 patients with head and neck cancer for this nationwide study using data from the Korean Health Insurance Review and Assessment Service. The patients were matched 1:5 with 60,635 control participants according to age, sex, and, region. Logistic regression analysis was used to estimate the odds ratios (ORs) and 95% confidence intervals (CIs) of cancer associated with β-blocker treatment. In the analysis, a crude (simple), adjusted model (adjusted model for age, sex, income, region of residence, hypertension, diabetes, and hyperlipidemia) was used.

The OR for head and neck cancer incidence was not lower in the β-blocker cohort (OR: 1.18; 95% CI: 1.105–1.26), especially for the oral cavity (OR: 1.165; 95% CI: 1.013–1.340), hypopharynx (OR: 1.555; 95% CI: 1.232–1.963), nasopharynx (OR: 1.251; 95% CI: 1–1.564), and paranasal sinus (OR: 1.378; 95% CI: 1.027–1.849). The duration of β-blocker use was not related to head and neck cancer incidence.

This study did not provide evidence that β-blockers can decrease the risk of head and neck cancer.

## 1 Introduction

β-blockers are used for various indications, particularly cardiac arrhythmias, cardioprotection after myocardial infarction, hypertension, migraine, and tremor, by inhibiting the sympathetic actions of catecholamine hormones (i.e., epinephrine and norepinephrine). These diverse indications reflect the abundance of β-adrenergic receptors in the body. β-blockers were prescribed more frequently for hypertension therapy in recent studies (10.8%, range 8–75%) than in previous studies.^[1]^ The prescription rate of secondary-prevention medications for post-acute coronary syndrome was 67.4% for β-blockers.^[2]^ Additionally, once β-blockers are prescribed, patients tend to use them for a long time.^[3]^

Experimental evidence shows that malignant cell lines express β-adrenergic receptors and that sympathomimetic neurotransmitters may affect carcinogenesis through these receptors.^[4–6]^ These neurotransmitters are suggested to have a major impact on secondary tumor growth and to contribute to metastasis, induction of angiogenesis mediated via vascular endothelial growth factor (VEGF) and interleukin (IL)-6, and tissue invasion.^[7–10]^

Given that β-blockers are considered safe, cheap, and effective, potential concomitant beneficial effects of their use for cancer would be of interest. However, evidence from epidemiological and clinical studies has been inconclusive. Several studies’ results have indicated that β-blockers could improve survival outcomes and reduce cancer risk, specifically of melanoma, ovarian, and prostate cancer.^[4,11–13]^ Others suggested that there is no meaningful evidence of an association between β-blocker use and cancer.^[14,15]^ However, data on the effect of β-blockers on head and neck cancer are sparse.^[11,12]^

We hypothesized that β-blockers could decrease the incidence of head and neck cancer. Therefore, we conducted a population-based case-control study to examine whether the use of β-blockers is associated with the incidence of head and neck cancer.

## 2 Materials and methods

### 2.1 Study population and data collection

During the study period, the Big Data Research Group of the Korean Society of Otorhinolaryngology–Head and Neck Surgery consistently reviewed and confirmed the results of the extracted data. The institutional review board of Korea University Ansan Hospital approved this study (Institutional Review Board no. AS16113).

The Korean National Health Insurance Service (NHIS) reported claims data of patients. We used the National Health Information Database (NHID) operated by the Korean NHIS, a government-affiliated agency under the Korean Ministry of Health and Welfare that administers and supervises all medical activities in Korea.^[16]^ All Korean citizens and registered foreigners, approximately 50,000,000 persons, are enrolled and receive medical services from the NHIS. Retrospective medical data for patients of all ages were extracted from the NHID from January 2006 to December 2015. The NHIS contains information on the patients’ demographics, medical service use, medication, transaction information, deductions, and claims. When a physician is consulted at a medical facility in the Republic of Korea, the physician is required to assign a code according to the most appropriate diagnosis. These codes must be based on the International Classification of Diseases, 10th edition (ICD-10), which is designed by the World Health Organization to efficiently manage diseases and health problems. Therefore, all such records of medical services conducted in the Republic of Korea will be assigned these diagnostic codes and stored in the NHID.

### 2.2 Participant selection

Based on the above information, a patient diagnosed at a hospital during the study period with a diagnostic code (ICD-10) for head and neck cancer (nasopharynx: C11; oral cavity: C00, 02–06; oropharynx: C01, 09, 10; hypopharynx: C12, 13; larynx: C32) was defined as a head and neck cancer patient. When the patient was also registered in the Korean cancer registration system, we regarded the patient as a head and neck cancer participant. All participants diagnosed accordingly (n = 12,127) were included in this study. Control participants (n = 60,635) were matched 1:5 according to age, sex, and region of residence. The systemic diseases investigated were hypertension (code I10 and received anti-hypertensive medication), diabetes (codes E11–14 with anti-diabetic medication), and hyperlipidemia (code E78). Ultimately, 12,127 head and neck cancer patients and 60,635 control subjects were enrolled in the study. Each cohort was also divided into 2 groups: β-blocker users and non-users. β-blocker users were defined as those who were prescribed β-blockers more than 2 times within 6 months before head and neck cancer registration. The index date was set as the initial date of the first treatment for prescriptions with more than 2 treatments. β-blocker non-users were defined as those who had not been prescribed β-blockers during the study period.

### 2.3 Statistical analyses

Statistical analyses were conducted using SAS software version 9.3 (SAS Institute, Cary, NC). Paired *t* test and Chi-square test were used to compare the general characteristics between the 2 groups. Logistic regression analysis was used to estimate the odds ratios (ORs) and 95% confidence intervals (CIs) of the incidence of head and neck cancers associated with β-blockers treatment. In this analysis, a crude (simple), adjusted model (adjusted model for age, sex, income, region of residence, hypertension, diabetes, and hyperlipidemia) was used. *P* values less than .05 indicated statistical significance.

## 3 Results

Age, sex, and region of residence were equivalent in both groups. In contrast, low income, comorbidities (hypertension, diabetes, and hyperlipidemia), and the use of β-blockers were significantly higher in the head-and-neck-cancer group than in the control group (Table 1). The risk of total head and neck cancer incidence was not lower in the β-blocker user group than in the β-blocker non-user group (OR: 1.180; 95% CI: 1.105–1.260). The duration of β-blocker use was not associated with the risk of total head and neck cancer (Table 2). Table 3 shows the incidences of the 7 head and neck cancer types (oral cavity, oropharynx, hypopharynx, larynx, salivary gland, nasopharynx, and paranasal sinus) according to the use of β-blockers and the duration of β-blocker use. Compared with the patients who did not use β-blockers, patients who underwent β-blocker treatment did not exhibit lower risks of cancer in the oral cavity (OR: 1.165; 95% CI: 1.013–1.340), hypopharynx (OR: 1.555; 95% CI: 1.232–1.963), nasopharynx (OR: 1.251; 95% CI: 1–1.564), and paranasal sinus (OR: 1.378; 95% CI: 1.027–1.849). In addition, the duration of β-blocker use was not related to the risk of the 7 head and neck cancer types.

**Table 1:**
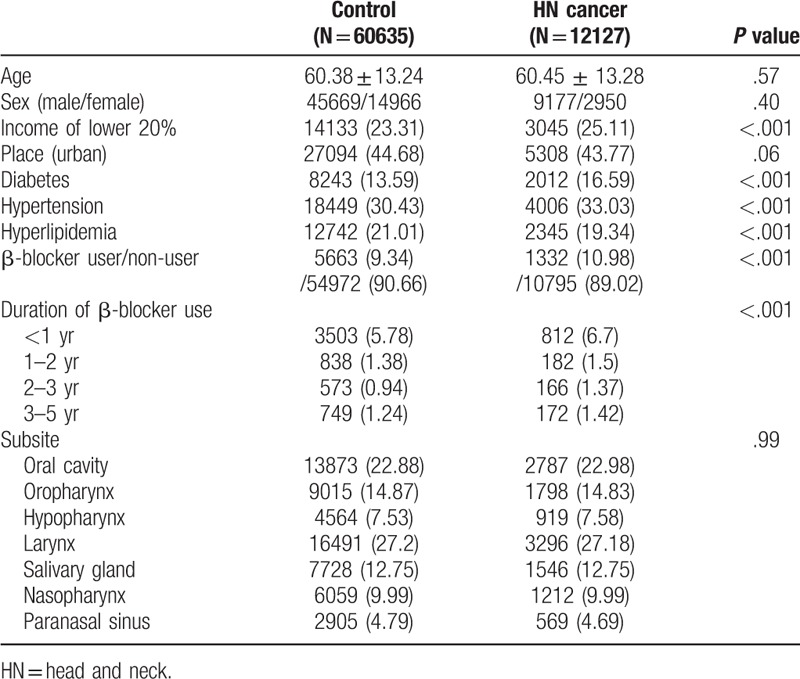
General characteristics of participants.

**Table 2:**
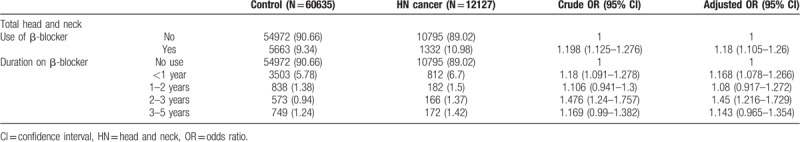
Incidence and adjusted odds ratios of total head and neck cancer stratified by duration of β-blocker use.

**Table 3:**
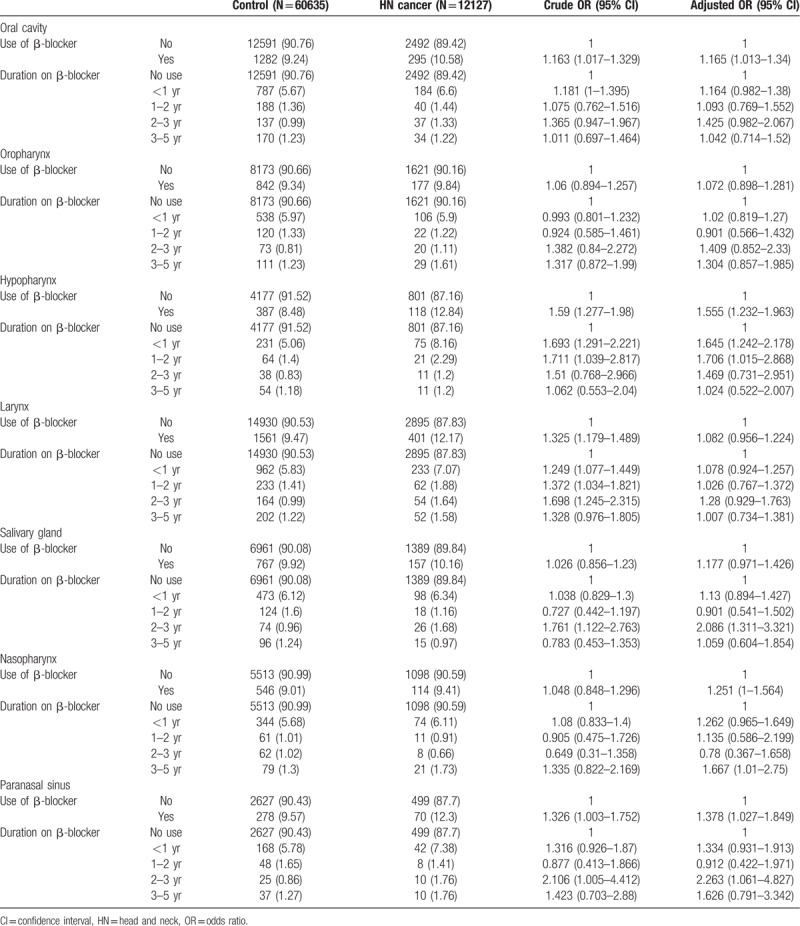
Incidence and adjusted odds ratios of specific head and neck cancer subsites stratified by duration of β-blocker use.

## 4 Discussion

We could not find a preventive effect of β-blockers for head and neck cancer. On the contrary, the risk of head and neck cancer incidence was not lower in β-blocker users than in β-blocker non-users. Additionally, the duration of β-blocker use was not associated with the risk of head and neck cancer. Moreover, β-blocker use did not lower cancer incidence in any head and neck cancer types. Thus, the hypothesis that β-blocker use, which interferes with the norepinephrine via blocking β-adrenergic receptor, decreases the risk of head and neck cancer could not be confirmed.

There have been some studies investigating the association between β-blockers and cancer risk. The results of one study on colorectal cancer were comparable to our findings since it did not show a beneficial effect of β-blocker use on cancer risk.^[17]^ A Taiwanese population-based cohort study showed that compared with patients who did not take propranolol, patients who received propranolol treatment did not exhibit significantly lower risks of cancer in the hepatobiliary tract (OR: 1.10; 95% CI: 0.82–1.47), lung (OR: 0.80; 95% CI: 0.58–1.10), skin (OR: 0.53; 95% CI: 0.22–1.24), breast (OR: 0.72; 95% CI: 0.50–1.02), uterus (OR: 0.60; 95% CI: 0.32–1.13), bladder (OR: 1.06; 95% CI: 0.58–1.92), kidney (OR: 1.71; 95% CI: 0.77–3.84), brain (OR: 0.27; 95% CI: 0.06–1.28), and thyroid (OR: 0.77; 95% CI: 0.30–1.94).^[11]^ Additionally, other studies found that the use of β-blockers in patients with cancer does not appear to have a consistent association with cancer recurrence or survival in either the epidemiological, clinical, or systematic review setting.^[4,14,15,18]^ However, the previously mentioned Taiwanese population-based cohort study showed that propranolol could decrease the risk of head and neck cancer, including 67 patients.^[11]^ Moreover, some clinical studies suggested that β-blocker use may be associated with improved survival outcomes in patients with cancer.^[12,13]^ Specifically, β-blocker use may be associated with better outcomes in specific types of cancer (e.g., melanoma and ovarian cancer), while an opposite effect was observed in patients with endometrial, prostate, or lung cancer. The lack of a causal relationship and specificity between β-blocker therapy and long-term cancer outcomes reflects the paucity of data but may also reflect the underlying heterogeneity in the response of cancer subtypes to β-blocker therapy modulation.^[14]^

β-adrenergic receptor expression is found on cancer and immune cells, and activation of these receptors in different cancer types has diverse effects on the tumor microenvironment (tumor proliferation, migration, and invasion). Importantly, in vivo studies that explored the effects of β-adrenergic receptor signals suggested a key role for the β2-adrenergic receptor in modulating tumor outcomes, and typically investigated β-blockades using propranolol, a non-selective beta-blocker.^[7–10]^ Some studies suggested that increased expression of β2-adrenergic receptor at the mRNA- and protein level in head and neck cancer cell lines may inhibit tumor proliferation.^[5,6,8]^ Nikolaus et al showed that propranolol reduced head and neck cancer viability, induced apoptosis, and inhibited the production of the proangiogenic protein VEGF.^[5]^ However, our study focused on whether pre-diagnostic use of β-blockers could lower head and neck cancer risk. In fact, it has been reported that post-diagnostic β-blocker use was associated with decreased survival in head and neck cancer.^[12]^ The results of a Taiwanese population-based cohort study indicated that propranolol can reduce the risk of head and neck cancer, which contrast with our results.^[11]^ However, the study analyzed only 67 patients with head and neck cancer, whereas this study investigated 12,127 head and neck cancer patients.

One strength of this study is the large number of study participants (N = 72,762). To our knowledge, this is the largest study that evaluated the relationship of β-blockers for head and neck cancer. In addition, few studies have been conducted according to specific head and neck cancer types. Another strength is the availability of comprehensive medical records for each participant. A previous study questioned participants about their history of β-blockers prescription, which could have introduced recall bias.^[12]^ In this study, we extracted patients’ medical records from the NHID regarding β-blockers treatment. These recorded data were not distorted by their memories. The NHID includes the entire population without exception. Therefore, we did not overlook any participants during the study period, while other studies were affected by significant losses during the study period.^[12]^ Although we did not use randomized controlled trial methods, we matched our participants with a control group according to age, sex, and region of residence. Region matching was important as it could be a determinant factor for access to medical treatment. Finally, our study results were based on data from the entire Korean population and were verified by a statistician for representativeness.

However, our study had several limitations. First, we used health insurance claims data, which may not have been reflective of the actual use of β-blockers by patients. However, medical claims data are very important in Korea. If the claim codes are incorrect, the medical claim fee cannot be paid by the NHIS. Moreover, β-blockers cannot be prescribed to patients without exact diagnosis codes, and patients could be rejected to private insurance services. Claim code data are not subject to recall bias; therefore, the associated medical data would be more accurate than if derived from surveys or subjective data from other studies. Moreover, the Korean cancer registration system compensates for the possibility of misdiagnoses, even though some patients were misdiagnosed. We also used a patient-control study design with a large number of participants, considering the possibility of misdiagnosis would exist in both the patient and control groups. Second, smoking is an important risk factor for head and neck cancers, and could, therefore, confound the association between beta-blockers and head- and neck cancer. However, this study did not investigate exactly head and neck cancer patients’ smoking history. Third, we could not extract all the information from the NHID because of security problems. Therefore, this study did not classify beta-blockers into several categories, such as non-selective, beta 1 selective, and beta 2 selective blocker. In addition, this study did not investigate the association between beta-blockers and cancer survival. Future prospective studies are needed to validate our findings. In conclusion, our study did not find a link between β-blocker use and a decrease in the incidence of head and neck cancer.

## Author contributions

The manuscript was edited for proper English language, grammar, punctuation, spelling, and overall style by the highly qualified native English-speaking editors at Editage.

**Conceptualization:** Soon Young Kwon.

**Data curation:** Kyung Do Han.

**Formal analysis:** Kyung Do Han.

**Funding acquisition:** Min-Su kim, Soon Young Kwon.

**Investigation:** Soon Young Kwon.

**Methodology:** Kyung Do Han.

**Software:** Kyung Do Han.

**Supervision:** Soon Young Kwon.

**Writing – original draft:** Min-Su kim.

**Writing – review & editing:** Min-Su kim.
